# Association between Red Blood Cell Distribution Width and Short-Term Mortality in Patients with Paralytic Intestinal Obstruction: Retrospective Data Analysis Based on the MIMIC-III Database

**DOI:** 10.1155/2023/6739136

**Published:** 2023-10-23

**Authors:** Xuelian Zhao, Xinhuan Wan, Chao Gu, Shanyu Gao, Jiahui Yin, Lizhu Wang, Longfang Quan

**Affiliations:** ^1^The First Clinical College, Shandong University of Traditional Chinese Medicine, Jinan 250013, Shandong Province, China; ^2^School of Pharmacy, Shandong University of Traditional Chinese Medicine, Jinan 250013, Shandong Province, China; ^3^Department of Anorectal, Affiliated Hospital of Shandong University of Traditional Chinese Medicine, Jinan 250014, Shandong Province, China; ^4^School of Traditional Chinese Medicine, Shandong University of Traditional Chinese Medicine, Jinan 250013, Shandong Province, China; ^5^Department of Anorectal, China Academy of Chinese Medical Sciences Xiyuan Hospital, Beijing 100091, China

## Abstract

**Objective:**

Elevated red cell distribution (RDW) has been reported to be associated with mortality in patients with acute pancreatitis and cholecystitis admitted to the intensive care unit (ICU). However, evidence for the relationship between RDW and paralytic intestinal obstruction is lacking. Therefore, the article aims to investigate the relationship between RDW and 28-day mortality of the patients with paralytic intestinal obstruction. *Patients and Methods*. This is a single-center retrospective study. Based on a particular screening criterion, 773 patients with paralytic intestinal obstruction were selected from the Medical Information Mart for Intensive Care III (MIMIC-III) database. Indicators of the first 24 h into the ICU were used to analyze the relationship between RDW and 28-day death from paralytic intestinal obstruction by Kaplan−Meier (K-M) analysis, logistic regression analysis, and stratification analysis.

**Results:**

The curve fitting exhibited a nonlinear relationship. The K-M curve showed that groups with higher RDW values had lower survival rates. The logistic regression analysis revealed that RDW increased with 28-day mortality in patients with paralytic intestinal obstruction in the fully adjusted model. In the fully adjusted model, OR value and 95% CI from the second to the third quantiles compared to the first quartile (reference group) were 1.89 (1.04, 3.44) and 3.29 (1.82, 5.93), respectively. The results of stratified analysis of each layer had the same trend as those of regression analysis, and the interaction results were not significant.

**Conclusion:**

Elevated RDW was associated with increased 28-day mortality from paralytic intestinal obstruction in the ICU. This study can help to further explore the relationship between RDW and death in patients with paralytic intestinal obstruction.

## 1. Introduction

In intensive care units (ICU), gastrointestinal failures, including paralytic intestinal obstruction, are common in patients [1]. Paralytic intestinal obstruction is a paralysis of the intestine without power, mainly caused by the disorder of the intestinal autonomic nervous system, block of local nerve conduction in the intestine, and obstruction of the contraction of the smooth muscle of the intestine, subsequently resulting in the powerlessness of dilation and peristalsis of the intestinal tube. If the diagnosis and treatment of intestinal obstruction are not timely, the disease can develop seriously and even induce intestinal necrosis, perforation, bacterial peritonitis, and other serious complications, consequently endangering the patients' lives [2].

Red cell distribution (RDW) is a routine blood test indicator, varying in value from 11% to 15%, which represents the degree of uniformity of red blood cell (RBC) size and can reflect the heterogeneity of erythrocyte volume in circulation [3]. RDW is an ideal laboratory test indicator and is also essential for the diagnosis and differential diagnosis of anemia due to its features of efficiency, effectivity, and low cost [4]. Studies have shown that the size of RDW is not only gradually increasing with age [5] but also positively correlated with HbA1c [6]. The risk factors of increased RDW include physical training, senility, erythropoietic stimulation, black race, and pregnancy [4]. Besides, the RDW index was reported to be related to the prognosis of some diseases, for instance, acute heart failure [7], cancer [8], acute kidney injury [3], and gastrointestinal disorders [9]. RDW is also an independent risk for mortality in the general population [4, 10, 11]. Moreover, RDW has also been linked to the death of patients with digestive-related illnesses in ICU, such as acute pancreatitis [12] and cholecystitis [13]. In ICU, the stratification of patients is more helpful to understand the prognosis of patients [14]. More and more literature has studied specific diseases and their specific indicators in ICU [12, 13, 15]. Doctors have a more accurate grasp of diseases and laboratory indicators, which is conducive to understanding the patients' conditions more accurately, having earlier communication with the patient's family members, relieving their anxiety, and providing favorable help for later diagnosis and treatment. In clinical studies, RDW has been proven to affect the prognosis of many diseases, but there is a lack of research on paralytic intestinal obstruction. Many studies confirmed that MIMIC-III plays an important role in clinical studies [16–19].

Studies have shown that the mortality rate of paralytic intestinal obstruction is 5%-6%, and the average cost of hospitalization is increasing [20]. Patients with paralytic intestinal obstruction in the ICU tend to have more severe symptoms, so short-term mortality is also increasing. Therefore, it is imperative to study the factors associated with the death of this disease. Inflammation plays a vital role in the progression of paralytic intestinal obstruction. In addition, some inflammation factors are to be associated with mortality from intestinal obstruction [21]. RDW has a high recognition ability for iron deficiency and iron deficiency anemia [22]. Serum ferritin is the most effective test for the diagnosis of iron deficiency. In addition, ferritin can be elevated in response to inflammation [22]. Therefore, our study aims to investigate whether RDW is associated with death in patients with paralytic intestinal obstruction after their entry into the ICU.

## 2. Materials and Methods

### 2.1. The Database

The retrospective cohort study explored the correlation between RDW and paralytic intestinal obstruction. All the data in this work were acquired from the Medical Information Mart for Intensive Care III (MIMIC-III). In this study, data analysis and publication comply with the statement of strengthening the reporting of observational studies in epidemiology. Jiahui Yin participated in the online course of the National Institutes of Health and passed the exam of the protection Human Research Participants (No. 40089742). Ultimately, she successfully accessed the MIMIC-III database, which is a unicentric and cost-free available database of multiple patients from the Beth Israel Deaconess Medical Center in Boston.

### 2.2. Inclusion and Exclusion Criteria for the Study Population

**Figure 1:**
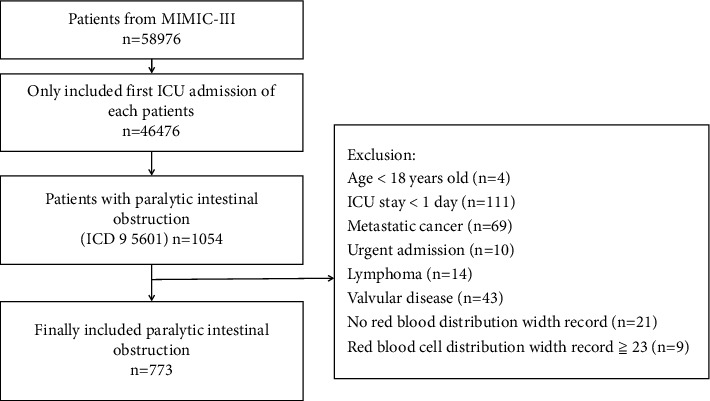
Flow diagram of selection for patients. Notes: In total, 58976 patients were included in the MIMIC-III. Records of 46476 first-time ICU admission were retained. Next, the patients with paralytic intestinal obstruction were extracted (*n* = 1054). Then, patients with the following standards were excluded: age <18-years old (*n* = 4), ICU stay <1 day (*n* = 111), metastatic cancer (*n* = 69), urgent admission (*n* = 10), lymphoma (*n* = 14), valvular disease (*n* = 43), no red blood distribution width record (*n* = 21), and red blood cell distribution width record ≥23 (*n* = 9). Finally, this study screened 773 patients for analysis. Abbreviations: MIMIC-III, medical information mart for intensive care III; ICU, intensive care unit; ICD, international classification of diseases.

The database contained an aggregate of 58,976 patients admitted to the ICU. In our research, 773 participants were selected through the screening criteria shown in Figure 1 for a flow chart. Patients over 18-years old with paralytic intestinal obstruction who were first hospitalized in the ICU for more than one day were enrolled. If patients had multiple hospital admissions in the database, we extracted the first admission. Patients with the following standards were excluded: (1) patients who miss RDW indicators; (2) patients with lymphoma, metastatic cancer, valvular disease, or emergency admission; (3) patients who miss over 5% individual data; (4) patients with RDW records more significant than or equal to 23%.

### 2.3. Outcome

The outcome measure was 28-day mortality after admission.

### 2.4. Data Extraction Method

PostgreSQL (version 9.6) was used to extract data from the database, including laboratory indicators, vital signs, and comorbidities.

### 2.5. Covariates

Demographic and admission information are as follows: age, sex, admission type, number of days in the hospital, admission time, quick Sequential Organ Failure Assessment score (qSOFA), Oxford Acute Severity of Illness Score (OASIS), Acute Physiology Score III (APS III), and Modified Logistic Organ Dysfunction System (MLODS).

### 2.6. Comorbidities

It includes congestive heart failure (CHF), liver disease, hypertension, renal failure, coagulopathy, chronic pulmonary disease, and pulmonary circulation.

### 2.7. Vital Signs

Systolic blood pressure (SBP) and diastolic blood pressure (DBP) were measured at ICU admission.

### 2.8. Laboratory Indicators

It includes white blood cell count (WBC), hematocrit, serum potassium, blood urea nitrogen (BUN), hemoglobin, platelet count, serum glucose, serum creatinine, and serum anion gap.

### 2.9. Statistical Methods

According to the tertiles of RDW, all the indicators were divided into three groups. The interquartile range (IQR) or mean (±SD) donated the dispersive or continuous variables, respectively. The number of cases (in percentages) represented the classification variables. Multivariable logistic regression models, crude model, minimally adjusted model, and fully adjusted model were used to test the independent effect of RDW on 28-day all-cause mortality. The minimally adjusted variables in the modified model consisted of sex, ethnicity, and age. The fully revised variables in the model included sex, ethnicity, age, SBP, DBP, BUN, WBC, serum glucose, creatinine, serum anion gap, APS III, OASIS, MLODS, liver disease, CHF, chronic pulmonary disease, and renal failure. Confounders were selected by the clinically significant results or *p* value less than 0.1 in the study population description or univariate analysis.

Next, subgroup analysis was performed to define the differences in RDW prognosis results in each subgroup. The Kaplan−Meier (K-M) survival curve was used to compare the patients' survival rates among three groups divided by the tertiles of RDW.

The data mentioned above, collation, and analysis were performed through statistical software package R and EmpowerStats software (https://www.empowerstats.com version R.3.6.3). *p* value less than 0.05 was considered statistical significance.

### 2.10. Ethical Considerations

The license was obtained by the Massachusetts Institute of Technology and the institutional review board of Beth Israel deacon Medical Center to establish the MIMIC-III database. Patients' informed consent was not required since none of the patients could be identified.

## 3. Results

### 3.1. Subject Characteristics

The diagram of selection for patients showed that 773 patients with paralytic intestinal obstruction were included in our study population. The features of the study population were shown in Table 1. There were three groups of RDW indicators as follows: the low group (12.0–14.1%), the middle group (14.2–15.7%), and the high group (15.8–23.0%). There was no difference in sex, serum potassium, WBC, serum glucose, qSOFA, chronic pulmonary disease, and time in ICU among low, middle, and high RDW. There was significance in age, admission type, ethnicity, SBP, DBP, hematocrit, BUN, platelet count, hemoglobin, serum creatinine, serum anion gap, APS III, OASIS, MLODS, CHF, hypertension, renal failure, liver disease, coagulopathy, pulmonary circulation disease, and time in hospital. In the scoring system, the values of APS III, OASIS, and MLODS increased with RDW, and the results showed obvious significance (*p*  <  0.05). Patients in the RDW high group had more comorbidities and higher values of MLODS, qSOFA, OASIS, and APSIII. Higher values of BUN, serum creatinine, and serum anion gap and lower values of hematocrit, WBC, hemoglobin, serum glucose, and platelet were observed in the high group. Increased 28-day mortality was noted in these patients.

### 3.2. Univariate Analysis

The results of the univariate analysis were shown in Table 2 in detail. Univariate analysis showed that qSOFA, congestive heart failure, liver disease, coagulopathy, and chronic pulmonary disease were positively correlated with 28-day mortality in patients with paralytic intestinal obstruction. In contrast, elective admission type was negatively associated with the mortality. There were indicators independent of 28-day mortality for patients with paralytic intestinal obstruction, including, DBP, serum potassium, BUN, WBC, platelet count, serum creatinine, serum anion gap, APS III, qSOFA, and MLODS.

### 3.3. RDW Levels and Mortality

**Figure 2:**
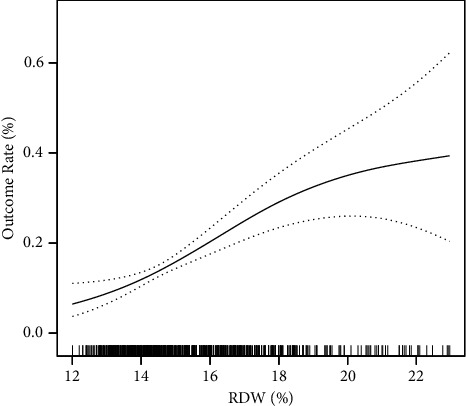
Association between RDW and 28 day mortality for patients with paralytic intestinal obstruction. Notes: A relationship between them was detected after adjusting for sex, ethnicity, age, SBP, DBP, BUN, WBC, serum glucose, creatinine, serum anion gap, APS III, OASIS, MLODS, congestive heart failure, chronic pulmonary disease, renal failure, and liver disease. A nonlinear relationship was found between RDW and paralytic intestinal obstruction mortality in the curve fitting diagram. The solid line in the middle represents the smooth curve fitting between variables. Imaginary lines represent the 95% of confidence interval from the fit. Abbreviations: RDW: red cell distribution width, BUN: blood urea nitrogen, SBP: systolic blood pressure, DBP: diastolic blood pressure, WBC: white blood cell, APS III: acute physiology score III, OASIS: Oxford acute severity of illness score, MLODS: modified logistic organ dysfunction system.

The curve fitting diagram is shown in Figure 2. The results of this figure showed a J-shape curve relationship between RDW and paralytic intestinal obstruction mortality. During the observation period, 127 (16.4%) participants died within 28-days after admission.  Table 3 is the logistic regression analysis. In the three models, the reference group was the low RDW (12.0–14.1). Logistic regression analysis further illustrated the relationship between RDW and short-term mortality in patients with paralytic intestinal obstruction. The crude model did not adjust for variables; the minimally adjusted model adjusted for age, sex and ethnicity, and the fully adjusted model adjusted for sex, ethnicity, age, SBP, DBP, BUN, WBC, serum glucose, creatinine, serum anion gap, APS III, OASIS, MLODS, CHF, chronic pulmonary disease, renal failure, and liver disease. It showed that an increase in RDW is associated with death from paralytic intestinal obstruction (OR 1.22, 95% CI 1.10, 1.35, *p*  <  0.001 in the minimally adjusted model; OR 1.22, 95% CI 1.10, 1.35, *p*  <  0.001 in the fully adjusted model). In addition, RDW was used as a categorical variable and the low RDW was the reference group. Middle RDW was associated with an increased short-term mortality in patients with paralytic intestinal obstruction compared with low RDW in the minimally adjusted model (OR 2.03, 95% CI 1.15, 3.60, *p*=0.015). High RDW was also associated with increased mortality in the minimally adjusted model (OR 3.90, 95% CI 2.27, 6.68, *p*  <  0.001). In the fully adjusted model, the relationship between the RDW and mortality was similar to that in the minimally adjusted model (OR 1.89, 95% CI 1.04, 3.44, *p*=0.036 in the middle RDW; OR 3.29, 95% CI 1.82, 5.93, *p*  <  0.001 in the high RDW). The *p* values of the trend tests are all less than 0.001 in all models.

### 3.4. Overall Survival

**Figure 3:**
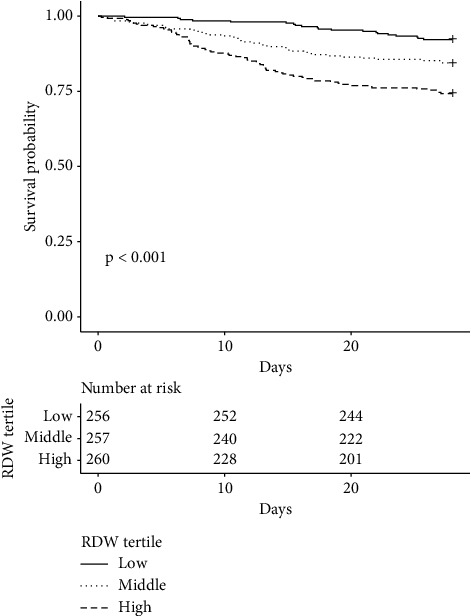
Overall survival. Notes: Kaplan−Meier curve presents cumulative survival to short-term mortality in the low group (12.0–14.1%), middle group (14.2–15.7%) and high group (15.8–23.0%) of the study population (*p*  <  0.001). At the same time point, patients in the higher RDW group had worse survival. Abbreviations: RDW: red cell distribution width.

The K-M curve displayed survival rates of subjects with different RDW levels. The K-M curves explained that patients in the RDW high group had shorter lifetime and higher death rates than those in the RDW medium and low groups (*p*  <  0.001) (Figure 3).

### 3.5. Subgroup Analysis

In this study, the following indicators were hierarchically analyzed: age, sex, admission type, ethnicity, renal failure, CHF, pulmonary circulatory disease, hypertension, chronic pulmonary disease, liver disease, and coagulopathy (Table 4). The trend of each subgroup was parallel to the regression analysis. No significant interaction was observed in different layers (*p*  >  0.05).

## 4. Discussion

Paralytic intestinal obstruction is a clinical syndrome in which intestinal motility dysfunction results in the inability to discharge the contents normally, often secondary to severe bowel surgery [23]. The disease not only delays the patient's discharge but also increases the risk of readmission. Although laxatives, cholinergic agonists, and other drugs can be used to treat this disease, there are still some side effects [24]. The mechanism responsible for the relationship between RDW and paralytic intestinal obstruction is unclear.

Previous studies have illustrated that the mortality during the progression of intestinal obstruction is related to inflammation indicated by many hematological parameters, such as WBC, neutrophile granulocyte [25], mononuclear leucocyte [21], C-reactive protein, erythrocyte sedimentation rate (ESR), and interleukin-6. These indicators are also prognostic characteristics of intestinal obstruction. For example, proinflammatory cytokines are associated with gastrointestinal motility retardation [26]. In addition, the preoperative stimulation of the vagus inhibiting inflammatory factors can alleviate postoperative intestinal obstruction [27]. Recently, it has been shown that RDW can serve as a marker of inflammatory response and a key indicator for diagnosing and observing the therapeutic effects of iron deficiency anemia [22]. Anemia resulting from systemic inflammatory response might exacerbate paralytic intestinal obstruction. Furthermore, patients with higher RDW values are more likely to have systemic symptoms and suffer more severe anemia, which leads to more serious diseases. The increase of RDW reflects the abnormality of RBC production, metabolism, and survival, caused by abnormal erythropoietin function, hypertension, oxidative stress, poor nutritional status, dyslipidemia, inflammation, RBC fragmentation, and shortened telomere length [4]. Accordingly, in addition to the inflammation marker, RDW may also be associated with death in patients with intestinal obstruction and could reflect microenvironment changes within the patients' bodies. Likewise, further investigation should be performed to examine the RDW's role in the mortality of paralytic intestinal obstruction.

The mortality of patients with paralytic intestinal obstruction can be affected by many factors. For example, a U-shape relationship between bicarbonate and mortality in ICU patients with paralytic intestinal obstruction was found [28]. In one study [29], fungal infection was associated with death in patients with pseudo-obstruction. Additionally, the high value of BUN at admission could lead to a poor prognosis in ICU patients [30]. At the same time, serum creatinine changes impacted on the outcomes of critically ill patients [31]. Studies have shown that many indicators can serve as risk factors for postoperative intestinal obstruction, including age, preoperative hemoglobin, operation time, American Society of Anesthesiologist score, surgical complexity, and RBC infusion [32]. For instance, age is positively correlated to RDW [10]. Gastrointestinal surgery is the most significant risk factor for paralytic intestinal obstruction. In addition, physical therapy can alleviate postoperative complications of emergency abdominal surgery, including paralytic intestinal obstruction [33]. Recently, it was reported that severe COVID-19 (coronavirus disease 2019) infection could also cause paralytic intestinal obstruction, which may be related to the virus entry mediated by the angiotensin-converting enzyme 2 receptor on cells in the gut and subsequent inflammation [34]. Additionally, RDW was found to be an independent risk factor by logistic regression analysis, which was also validated by substantive clinical observation. This finding applies to the guidance of clinical treatment. RDW is generally improved by defective hematopoietic material or increased RBC destruction. High baseline RDW is significantly associated with hemoglobin, bone marrow plasma cell infiltration, and cytogenetic risk stratification [35]. Past studies showed a link between RDW and mortality from digestive diseases in the ICU [12, 13]. However, the role of RDW in the 28-day mortality of paralytic intestinal obstruction is still unclear. Therefore, it is worth exploring the relationship between short-term mortality and RDW index changes in patients with paralytic intestinal obstruction.

The purpose of this study is to investigate the relationship between RDW and mortality in patients with paralytic intestinal obstruction. Our study found that the short-term mortality after admission in patients with paralytic intestinal obstruction increased with RDW. In this study, patients with metastatic cancer [36], lymphoma [37], valvular disease, and urgent admission [5] were excluded due to the independence of RDW to the survival time of these diseases. A previous retrospective study [13] on the ICU showed an increase in short-30-day mortality in patients with cholecystitis with increasing RDW (HR 1.183, 95% CI 1.080, 1.295). This analysis observed a nonlinear relationship between changes in RDW and 28-day all-cause mortality in patients with paralytic intestinal obstruction. The study in this paper also had a similar trend, and the difference in effect size between the two was not significant. It may be because both of them were digestive system diseases, so the two directions were similar. In the K-M curve, we observed that higher RDW levels had lower survival rates than lower RDW levels. Multiple regression equations indicated that an increase in RDW was associated with an increase in 28-day mortality in patients with paralytic intestinal obstruction after admission to the ICU. In addition, after adjusting for confounding factors, the link remained. High levels of RDW were significantly associated with mortality in these patients. Therefore, this study can help medical staff to explore further the relationship between RDW and death in patients with paralytic intestinal obstruction. In each stratum of the subgroup analysis, increased RDW was associated with increased mortality in patients with paralytic intestinal obstruction. In addition, the interaction results were not significant. Therefore, stratified analysis results excluded CHF, hypertension, chronic pulmonary disease, renal failure, liver disease, and coagulopathy on mortality. All these results suggested that the relationship between RDW and 28-day mortality in patients with paralytic intestinal obstruction in the ICU is stable. Paralytic intestinal obstruction can cause or aggravate malnutrition, water, and electrolyte imbalance, which further increases the risk of severe systemic infection, bleeding, organ failure, and indirectly increases the risk of death.

In the present work, a strong association between RDW changes and the mortality of patients with paralytic intestinal obstruction was found and fully supported by substantial data analysis, which is vital for the short-term prognosis of patients with paralytic intestinal obstruction. However, there are still some limitations to this study. First, conclusions drawn from this work cannot be extrapolated to people with metastatic cancer, urgent admission, lymphoma, valvular disease, or RDW record ≥23, due to the exclusion criteria. Second, since the database of this study is from the United States, the results of this study are not applicable to other populations. Data from different regions should have been included. Third, RDW is a time-varying variable and its causal associations with the outcome should be explored with more advanced techniques [38]. But only a single measure of RDW was used in this work, so there is a lack of research on causality. Fourth, the data of this study were obtained from the MIMIC database, which needs to be verified by more ICU databases. Fifth, laboratory indicators of some patients are missing or abnormal. Sixth, since this database has been continuously updated, new databases are needed for continuous verification to ensure the accuracy of experimental results. A possible next step would be to conduct additional research in MIMIC-IV.

## 5. Conclusion

Through analysis of the MIMIC database, we found that RDW was associated with 28-day of admission to the ICU in patients with paralytic intestinal obstruction. More research is needed to explore further the relationship between the two.

## Figures and Tables

**Table 1:** Baseline characteristics of the study population.

Characteristic	RDW (%) tertile			*p* value
	Low (12.0–14.1)	Middle (14.2–15.7)	High (15.8–23.0)	
Number, *n*	256	257	260	
RDW start	13.5 (0.5)	14.9 (0.4)	17.7 (1.7)	<0.001
Age (years)	60.4 (17.4)	65.7 (15.7)	62.3 (14.7)	<0.001
Sex, *n* (%)				0.797
Male	166 (64.8)	168 (65.4)	163 (62.7)	
Female	90 (35.2)	89 (34.6)	97 (37.3)	
Admission type, *n* (%)				0.003
Emergency	192 (75.0)	174 (67.7)	210 (80.8)	
Elective	64 (25.0)	83 (32.3)	50 (19.2)	
Ethnicity, *n* (%)				0.047
White	183 (71.5)	201 (78.2)	181 (69.6)	
Asian	12 (4.7)	6 (2.3)	4 (1.5)	
Black	25 (9.8)	15 (5.8)	31 (11.9)	
Others	36 (14.1)	35 (13.6)	44 (16.9)	
SBP (mmHg)	120.8 (17.1)	117.9 (16.7)	115.4 (16.8)	0.002
DBP (mmHg)	63.7 (10.4)	60.4 (10.0)	59.8 (10.4)	<0.001
Laboratory parameters				
Hematocrit (%)	33.3 (5.4)	31.4 (4.8)	29.6 (4.7)	<0.001
Serum potassium (mEq/L)	4.1 (0.6)	4.2 (0.6)	4.2 (0.6)	0.542
BUN (mg/dL)	17.0 (12.9–25.9)	21.0 (14.0–32.3)	26.2 (16.0–44.5)	<0.001
White blood cell count (K/uL)	11.2 (8.9–15.2)	11.5 (8.3–15.5)	10.1 (6.3–16.9)	0.388
Platelet count (K/uL)	208.5 (160.6–269.2)	209.0 (142.0–286.0)	177.8 (94.2–272.4)	0.009
Hemoglobin (g/dL)	11.4 (1.9)	10.6 (1.7)	9.9 (1.6)	<0.001
Serum glucose (mg/dL)	142.4 (46.4)	139.9 (66.4)	137.0 (45.8)	0.526
Serum creatinine (mg/dL)	0.9 (0.8–1.2)	1.1 (0.8–1.7)	1.2 (0.8–2.1)	<0.001
Serum anion gap (mEq/L)	13.2 (3.3)	13.6 (3.3)	15.0 (4.1)	<0.001
Scoring systems				
APS III	43.4 (20.3)	50.3 (21.5)	58.5 (22.6)	<0.001
OASIS	32.1 (9.2)	33.6 (8.7)	34.7 (9.1)	0.003
qSOFA	1.8 (0.7)	1.8 (0.7)	1.9 (0.7)	0.218
MLODS	2.0 (1.0–4.0)	3.0 (2.0–5.0)	4.0 (2.0–5.0)	<0.001
Comorbidities				
Congestive heart failure, *n* (%)				<0.001
No	225 (87.9)	210 (81.7)	180 (69.2)	
Yes	31 (12.1)	47 (18.3)	80 (30.8)	
Hypertension, *n* (%)				0.004
No	238 (93.0)	223 (86.8)	217 (83.5)	
Yes	18 (7.0)	34 (13.2)	43 (16.5)	
Renal failure, *n* (%)				<0.001
No	237 (92.6)	218 (84.8)	204 (78.5)	
Yes	19 (7.4)	39 (15.2)	56 (21.5)	
Liver disease, *n* (%)				<0.001
No	249 (97.3)	237 (92.2)	201 (77.3)	
Yes	7 (2.7)	20 (7.8)	59 (22.7)	
Coagulopathy, *n* (%)				<0.001
No	231 (90.2)	212 (82.5)	162 (62.3)	
Yes	25 (9.8)	45 (17.5)	98 (37.7)	
Chronic pulmonary disease, *n* (%)				0.545
No	200 (78.1)	205 (79.8)	197 (75.8)	
Yes	56 (21.9)	52 (20.2)	63 (24.2)	
Pulmonary circulation disease, *n* (%)				0.008
No	246 (96.1)	241 (93.8)	232 (89.2)	
Yes	10 (3.9)	16 (6.2)	28 (10.8)	
Time in ICU (days)	3.8 (2.0–9.4)	3.8 (2.0–9.0)	4.0 (2.0–8.1)	0.504
Time in hospital (days)	13.3 (9.2–22.0)	14.7 (9.9–23.9)	17.9 (10.5–32.0)	<0.001

Notes: normally distributed data are presented as the mean (SD) (analysis of variance); nonnormally distributed data are presented as median (IQR) (nonparametric Wilcoxon test); categorical variables are presented as *n* (%) (Chi-square test). Abbreviations: RDW, red cell distribution width; BUN, blood urea nitrogen; SBP, systolic blood pressure; DBP, diastolic blood pressure; APS III, acute physiology score III; OASIS, Oxford acute severity of illness score; qSOFA, quick sequential organ failure assessment score; MLODS, modified logistic organ dysfunction system; ICU, intensive care unit; SD, standard deviation; IQR, interquartile ranges.

**Table 2:** Univariate analysis for 28-day mortality in critically ill patients with paralytic intestinal obstruction.

Characteristic	Statistics	OR (95%CI)	*p* value
RDW start	15.4 (2.1)	1.25 (1.15, 1.36)	<0.001
Age (years)	62.8 (16.1)	1.02 (1.01, 1.03)	0.001
Sex, *n* (%)			
Male	497 (64.3)	Reference	
Female	276 (35.7)	0.99 (0.66, 1.47)	0.944
Admission type, *n* (%)			
Emergency	576 (74.5)	Reference	
Elective	197 (25.5)	0.72 (0.45, 1.14)	0.158
Ethnicity, *n* (%)			
White	565 (73.1)	Reference	
Asian	22 (2.8)	0.78 (0.23, 2.69)	0.696
Black	71 (9.2)	0.81 (0.40, 1.64)	0.56
Others	115 (14.9)	0.98 (0.57, 1.68)	0.939
DBP (mmHg)	61.3 (10.4)	0.99 (0.97, 1.01)	0.212
SBP (mmHg)	118.0 (17.0)	0.98 (0.97, 1.00)	0.014
Laboratory parameters			
Hematocrit (%)	31.4 (5.2)	0.95 (0.91, 0.99)	0.015
Serum potassium (mEq/L)	4.2 (0.6)	0.91 (0.65, 1.26)	0.553
BUN (mg/dL)	20.5 (14.0–34.0)	1.00 (0.99, 1.01)	0.548
White blood cell count (K/uL)	11.0 (8.1–16.0)	1.00 (0.98, 1.02)	0.899
Platelet count (K/uL)	202.0 (137.0–275.0)	1.00 (1.00, 1.00)	0.077
Hemoglobin (g/dL)	10.6 (1.8)	0.83 (0.74, 0.94)	0.002
Serum glucose (mg/dL)	139.8 (53.7)	0.99 (0.99, 1.00)	0.009
Serum creatinine (mg/dL)	1.0 (0.8–1.6)	0.99 (0.87, 1.14)	0.902
Serum anion gap (mEq/L)	13.9 (3.7)	1.01 (0.96, 1.07)	0.577
Scoring systems			
APS III	50.8 (22.3)	1.01 (1.00, 1.02)	0.059
OASIS	33.5 (9.1)	1.04 (1.02, 1.06)	<0.001
qSOFA	1.8 (0.7)	1.20 (0.91, 1.58)	0.191
MLODS	3.0 (1.0–5.0)	1.07 (1.00, 1.14)	0.068
Comorbidities			
Congestive heart failure, *n* (%)			
No	615 (79.6)	Reference	
Yes	158 (20.4)	1.96 (1.28, 3.01)	0.002
Hypertension, *n* (%)			
No	678 (87.7)	Reference	
Yes	95 (12.3)	0.86 (0.47, 1.58)	0.635
Renal failure, *n* (%)			
No	659 (85.3)	Reference	
Yes	114 (14.7)	1.10 (0.65, 1.86)	0.728
Liver disease, *n* (%)			
No	687 (88.9)	Reference	
Yes	86 (11.1)	2.05 (1.22, 3.45)	0.007
Coagulopathy, *n* (%)			
No	605 (78.3)	Reference	
Yes	168 (21.7)	1.95 (1.28, 2.96)	0.002
Chronic pulmonary disease, *n* (%)			
No	602 (77.9)	Reference	
Yes	171 (22.1)	1.57 (1.02, 2.41)	0.038
Pulmonary circulation, *n* (%)			
No		Reference	
Yes	54 (7.0)	1.17 (0.57, 2.39)	0.668
Time in ICU (days)	3.9 (2.0–8.7)	1.03 (1.01, 1.04)	0.76
Time in hospital (days)	15.1 (9.8–25.0)	1.02 (1.01, 1.03)	<0.001

Notes: normally distributed data are presented as the mean (SD) (analysis of variance); non-normally distributed data are presented as median (IQR) (nonparametric Wilcoxon test); categorical variables are presented as *n* (%) (Chi-square test). Abbreviations: RDW, red cell distribution width; BUN, blood urea nitrogen; SBP, systolic blood pressure; DBP, diastolic blood pressure; APS III, acute Physiology Score III; OASIS, Oxford acute severity of illness score; qSOFA, quick sequential organ failure assessment score; MLODS, modified logistic organ dysfunction system; ICU, intensive care unit; SD, standard deviation; IQR, interquartile ranges.

**Table 3:** OR (95% CI) for 28-day mortality across groups of RDW.

Exposure	Crude model	Minimally adjusted model	Fully adjusted model
Clinical parameters, *n*	773	773	761
RDW start	1.25 (1.15, 1.36)	1.25 (1.15, 1.37)	1.22 (1.10, 1.35)
	<0.001	<0.001	<0.001
Per 1 SD	1.60 (1.35, 1.90)	1.60 (1.34, 1.91)	1.51 (1.23, 1.85)
	<0.001	<0.001	<0.001
RDW tertile			
Low	Reference	Reference	Reference
Middle	2.18 (1.23, 3.84)	2.03 (1.15, 3.60)	1.89 (1.04, 3.44) 0.036
	0.007	0.015	
High	4.10 (2.40, 6.99)	3.90 (2.27, 6.68)	3.29 (1.82, 5.93)
	<0.001	<0.001	<0.001
*P* for trend	<0.001	<0.001	<0.001

Notes: For the crude model, we did not adjust other covariants. For the minimally adjusted model, we adjusted age, sex, and ethnicity. For the full adjusted model, we adjust sex, ethnicity, age, SBP, DBP, BUN, WBC, serum glucose, creatinine, serum anion gap, APS III, OASIS, MLODS, congestive heart failure, chronic pulmonary disease, renal failure, and liver disease. Abbreviations: CI, confidence interval; OR, odds ratio; RDW, red cell distribution width; SD, standard deviation.

**Table 4:** Effect size of RDW on 28 day mortality in prespecified and exploratory subgroups.

Characteristic	No of participants	OR (95%CI)	*P* for interaction
Age (years)			0.791
≤65	370	1.25 (1.09, 1.42)	
>65	403	1.28 (1.14, 1.42)	
Sex, *n*			0.571
Male	497	1.28 (1.15, 1.42)	
Female	276	1.22 (1.06, 1.40)	
Admission type, *n*			0.282
Emergency	576	1.28 (1.16, 1.40)	
Elective	197	1.13 (0.91, 1.39)	
Ethnicity, *n*			0.277
White	565	1.23 (1.12, 1.36)	
Asian	22	2.40 (0.96, 6.01)	
Black	71	1.42 (1.08, 1.88)	
Others	115	1.21 (0.99, 1.49)	
Congestive heart failure, *n*			0.355
No	615	1.27 (1.15, 1.39)	
Yes	158	1.15 (0.96, 1.38)	
Hypertension, *n*			0.751
No	678	1.26 (1.15, 1.38)	
Yes	95	1.31 (1.03, 1.66)	
Renal failure, *n*			0.263
No	659	1.23 (1.12, 1.35)	
Yes	114	1.40 (1.14, 1.72)	
Liver disease, *n*			0.481
No	687	1.26 (1.14, 1.39)	
Yes	86	1.16 (0.96, 1.41)	
Coagulopathy, *n*			0.184
No	605	1.28 (1.15, 1.43)	
Yes	168	1.13 (0.97, 1.31)	
Chronic pulmonary disease, *n*			0.995
No	602	1.26 (1.14, 1.38)	
Yes	171	1.26 (1.05, 1.50)	
Pulmonary circulation, *n*			0.656
No	719	1.27 (1.16, 1.38)	
Yes	54	1.19 (0.92, 1.54)	

Notes: things presented in this table are results for unadjusted variables. Abbreviations: DW, red cell distribution width; CI, confidence interval; OR, odds ratio.

## Data Availability

The datasets generated during the current study are available in the MIMIC-III repository (https://mimic.mit.edu/).
